# Detecting Hospital Outliers in Post-Pancreatectomy Care Using Funnel Plots from 2009–2018 Based on Nationwide Medico-Administrative Data

**DOI:** 10.1007/s00268-021-06078-4

**Published:** 2021-04-05

**Authors:** Alain Bernard, Jonathan Cottenet, Serge Aho, Alexandre Doussot, Anne-Sophie Mariet, Olivier Facy, Catherine Quantin

**Affiliations:** 1 Department of Thoracic and Cardiovascular Surgery, Dijon University Hospital, CHU Dijon, Hôpital du Bocage, 14 rue Gaffarel, BP 77908, 21079 Dijon, France; 2 Department of Biostatistics, Dijon University Hospital, University of Burgundy, Dijon, France; 3 Bacteriology and Hospital Hygiene Department, Nantes University Hospital, Nantes, France; 4 Department of Digestive Surgery, Dijon University Hospital, Dijon, France; 5 INSERM, CIC 1432, Clinical Investigation Centre, Dijon University Hospital, University of Burgundy, Dijon, France; 6 INSERM, UVSQ, Pasteur Institute, Paris-Saclay University, Paris, France

## Abstract

**Objectives:**

Our objective was to identify hospitals with unusual mortality rates for major pancreatectomies over a period of ten years using 30-day mortality data from the French national database.

**Methods:**

Data for all patients who underwent pancreatectomy were extracted from the national medico-economic database (*Programme de Médicalisation des Systèmes d'Information*). To identify quality outliers for each hospital, the observed-to-expected 30-day mortality rates were used as a quality indicator.

**Results:**

A total of 19 494 patients underwent a major pancreatectomy in France between January 2009 and December 2018. The overall 30-day mortality rate was 4.8% (*n* = 944). For the 2009–2014 period, the funnel plot showed that 10 of the 176 hospitals lie outside the central 95% region and 7 lie outside the central 99.8% region. For the 2015–2018 period, out of 176 hospitals, 6 lie outside the central 95% region and 2 lie outside the central 99.8% region. The change in standardized mortality ratios between 2009–2014 and 2015–2018 testing for differences from the overall change, they were there 4 hospitals lie outside the central 95% region and 0 lie outside the central 99.8% region.

**Conclusion:**

Over time, the improvement in hospital quality was weak. This study suggests that there is a pressing need to reorganize the supply of care for pancreatic surgery in France.

## Introduction

Pancreatectomy requires expertise both for the procedure and for the management of postoperative complications [1–4]. For these reasons, many countries have reorganized care by proposing a system of regionalization [5, 6]. The idea is to group the appropriate teams on the same site, resulting in an increase in the volume of activity. While numerous studies have shown that hospital volume significantly influences the risk of postoperative death, the volume of activity alone is not enough to measure hospital performance [4–6].

In France, despite the availability of medico-administrative data, there is a paucity of literature relative to the surgical performance of hospitals and clinics. Meanwhile, the vast majority of the French population has direct access to health care, which makes it possible to investigate the practice of pancreas surgery through an assessment of quality of care from the national medico-administrative database. To identify hospitals with standard performance, standardized mortality ratios (SMRs) have been used as performance indicators [7–9]. As a commonly used metric, the 30-day mortality rate captures most surgery-related deaths for pancreatic surgery [7], therefore representing a legitimate measure of surgical quality. Here, we are interested in identifying quality outlier hospitals using funnel plots [10]. Using a national database, we aimed to analyze the change over time in the performance of hospitals that perform major pancreatectomies.

The objective of this study was to use the national medico-administrative database to determine hospital performance for major pancreatectomies that deviated from the national 30-day mortality rate from as an indicator of performance from 2009–2018.

## Material and methods

### Data source and study population

Data for all patients who underwent pancreatectomy in France between 2009 and 2018 were extracted from the national medico-administrative database (*Programme de Médicalisation des Systèmes d'Information* (PMSI)). We used the 10th revision of the International Classification of Diseases (ICD-10) to identify relevant diagnosis codes in the discharge abstracts [11–13]. Patients were selected when the primary diagnosis was a malignant tumor (all C25 codes) or benign tumor (D136, D137, K868**)**. The Common Classification of Medical Procedures (CCAM) was used to define relevant interventions: pancreaticoduodenectomy and total pancreatectomy.

### Patient characteristics

In addition to collecting data for age and sex, we used the ICD-10 codes to identify comorbidities present at the time of hospitalization: pulmonary disease (chronic bronchitis, emphysema), heart disease (coronary artery disease, arrhythmia, chronic heart failure, valvulopathy, pulmonary embolism), peripheral vascular disease (aneurysm, peripheral vascular disease), neurological disease (stroke, neurological sequelae, dementia), liver disease, renal disease, anemia, infectious disease, hematologic disease and other treatments (neo-adjuvant chemotherapy or corticosteroid therapy). We calculated a modified Charlson Comorbidity Index (CCI) score for each patient [14].

Patient consent was not required. Ethics approval for use of this database was obtained from the French National Commission for Data protection (*Commission Nationale de l’Informatique et des Libertés*: No 1576793), and this study adhered to the tenets of the Declaration of Helsinki.

### Hospital characteristics

Hospitals were classified as non-teaching hospitals, private for-profit institutions, private non-profit institutions or teaching hospitals. The annual volume of each establishment was estimated.

### Outcome measurements

30-day mortality was defined as any death occurring within 30 days of surgery or during the same hospitalization as pancreatectomy.

### Statistical methods

#### Risk-adjustment model

We performed univariate analyses with Chi-squared tests for binary and categorical variables and Student’s *t* tests for continuous variables. Logistic regression models were constructed for the 2009–2014 period using backward stepwise variable selection for comorbidities. We used a bootstrap backward procedure, as recommended by Steyerberg [15], to determine which of these factors were significantly associated with the outcome in logistic regression models. Using this approach, 1000 replicated bootstrap samples were selected from the original data. In each replicated sample, variables such as age, gender and modified Charlson Comorbidity Index score were forced into the model. Risk factors selected in at least 500 samples (50%) of the replicates were included in the model. We then included the variables for hospital characteristics (volume and type of establishment) in the model. The hospital volume was a continuous variable that was transformed into a logarithm to be included in the model.

We did the following to assess the quality of our model [15]: the area under the receiver operating characteristic (ROC) curve was used to measure the discriminatory ability [15] and the Hosmer–Lemeshow goodness-of-fit test to assess the reliability of the model.

For the 2015–2018 period, we updated the model by testing three methods described by Steyerberg [15]: calibration-in-large, recalibration and model revision.

#### Identification of quality outliers

For the analysis of hospital outliers over time, we grouped the years together as follows: 2009–2014 and 2015–2018. Observed-to-expected rates (O/E ratio) can be used as a quality indicator for hospitals. The O/E ratio is observed mortality divided by the expected mortality rate estimated from a logistic regression model. For each period, we constructed funnel plots to determine outliers for 30-day mortality according to Spiegelhalter’s methodology [16] To calculate an overdispersion factor for risk adjusted rates, we used a multiplicative approach with a Winsorized estimator (10th and 90th percentiles) [17]. We used funnel plots to evaluate changes in the standardized mortality ratio (SMR) between 2009–2014 and 2015–2018, and we calculated the SMR1 for 2015–2018 divided by the SMR2 for 2009–2014 [16]. The ratio (SMR2/SMR1) was then transformed into a logarithm.

Calculations were performed with STATA 14 statistical software (StataCorp, College Station, Tex) and R statistical software (http://www.r-project.org).

## Results

**Fig. 1:**
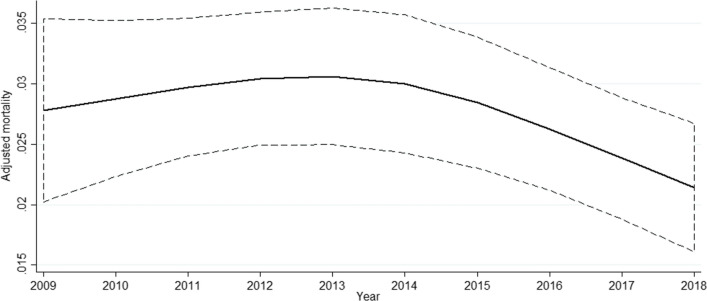
Change in the 30-day mortality rate over time

A total of 19 494 patients underwent major pancreatectomy in France between January 1, 2009 and December 31, 2018. The 30-day mortality rate was 4.84% (*n* = 944). The mortality rate decreased from 2014, as shown in Fig. 1. The creation of two periods going from 2009–2014 and 2015–2018 is justified by the change in the mortality rate (Fig. 1). Patient characteristics are presented in Table 1. Mean patient age increased significantly during the second period, and comorbidities such as metabolic disease, anemia and infectious disease were also more frequent during the second period (Table 1). The modified Charlson Comorbidity Index score was higher during the second period. During the two periods, 176 hospitals performed major pancreatectomies. The median number of pancreatic resections per year was 5 for the first period and 7 for the second period (Table 1).

**Table 1:** Patient characteristics and hospital structure according to period

	2009–2014 (*n* = 10 739)	2015–2018 (*n* = 8 755)	*p* value
Age (years)	62.7 ± 13	64 ± 13	0.0001
*Sex*			
Male	5 579 (52%)	4 533 (52%)	0.8
Female	5 160 (48%)	4 222 (48%)	
*Comorbidities*			
Pulmonary disease	1 406 (13%)	1 229 (14%)	0.05
Heart disease	1 330 (12%)	1 075 (12%)	0.8
Peripheral vascular disease	682 (6%)	596 (7%)	0.2
Neurological disease	311 (3%)	279 (3%)	0.2
Liver disease	256 (2%)	205(2%)	0.8
Renal disease	224(2%)	177(2%)	0.7
Metabolic disease	2603 (24%)	2 304(26%)	0.001
Anemia	3 194 (30%)	3 443(39%)	0.0001
Infectious disease	303(3%)	413(5%)	0.0001
Hematologic disease	835(8%)	728(8%)	0.17
Other diseases	4 360 (41%)	4 011(46%)	0.0001
Other treatment	1 086 (10%)	955 (11%)	0.07
*Modified Charlson Comorbidity Index Score*			
0 or 1	1 866 (17%)	1 242 (14%)	0.0001
2	4 214 (39%)	3 272 (37%)	
≥ 3	4 659 (43%)	4 241 (48%)	
30-day mortality	544 (5%)	400 (4.5%)	0.1
*Hospitals*			
Non-teaching	1 180 (11%)	1 016 (12%)	0.14
Private non-profit	1 372 (13%)	1 042 (12%)	
Private for-profit	2 770 (25%)	2 219 (25%)	
Teaching	5 417 (50%)	4 478 (51%)	
*Hospital volume*			
Number of procedure per year^a^	5 (3–10)	7 (4–12)	0.2

^*a*^median (interquartile)

### Risk-adjustment models

The model developed during the first period is reported in Table 1. This model had good performance with a C-index of 0.814 (Table 2). The Hosmer–Lemeshow goodness-of-fit test was nonsignificant for this model (Chi^2^ 9.4, *p* < 0.3) (Table 1). For the second period, we were forced to use a revised model to obtain good performance with a C-index of 0.805 (Table 2). The Hosmer–Lemeshow goodness-of-fit test was nonsignificant for this model (Chi^2^ 9.8, *p* < 0.28) (Table 2). It should be noted that the kidney disease variable had more weight in the revised model (Table 2).

**Table 2:** Logistic regression coefficients developed in period 2009–2014 and updated revised model in period 2015–2018

	2009–2014 Original model	2015–2018 Revised model	*p* value
Female	−0.2059	−0.5405	0.0001
Age	0.0432	0.038	0.03
Pulmonary disease	1.1012	1.1443	0.0001
Heart disease	0.5313	0.1475	0.001
Peripheral vascular disease	0.3883	0.2262	0.009
Liver disease	2.4504	2.5743	0.0001
Renal disease	0.771	1.3057	0.0002
Metabolic disease	−0.3407	−0.3273	0.0047
Anemia	−0.3046	−0.1977	0.0043
Infectious disease	0.7524	0.6356	0.0001
Other disease	0.2708	0.3426	0.01
*CCI score*			
2	1.3308	0.6681	0.0001
≥ 3	1.3796	0.679	0.0001
*Hospital*			
Private non-profit	−0.1765	−0.6125	0.44
Private for-profit	0.5362	−0.1232	0.0012
Teaching	0.4887	−0.4965	0.02
Logarithm number of procedure per year	−0.2411	−0.1139	0.0005
Intercept	−7.2326	−5.9394	
R2	0.218	0.201	
Brier scale	0.04	0.04	
C-Statistic	0.814	0.805	
Hosmer–Lemeshow test (*p* value)	9.4 (0.3)	9.8 (0.27)	

### Identification of quality outliers

**Fig. 2:**
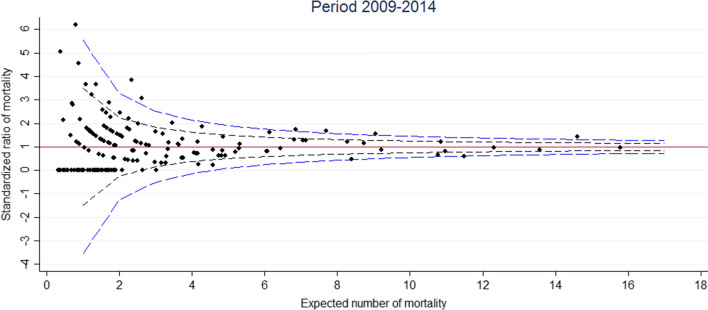
Standardized ratio of mortality against expected number of mortality. Funnel plot with band limits at 95% (black dash line) and 99.8% (blue dash line) during the 2009–2014 period

**Fig. 3:**
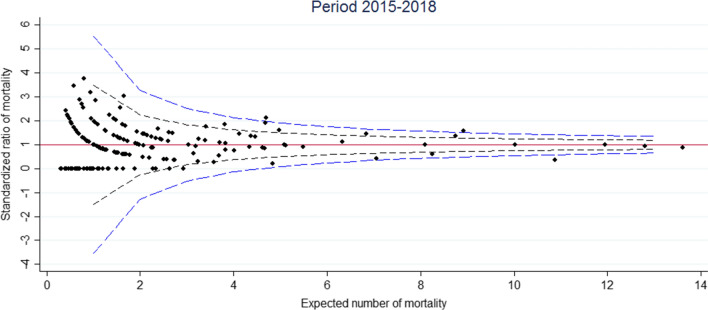
Standardized ratio of mortality compared to expected mortality. Funnel plot with band limits at 95% (black dash line) and 99.8% (blue dash line) during the 2015–2018 period

**Fig. 4:**
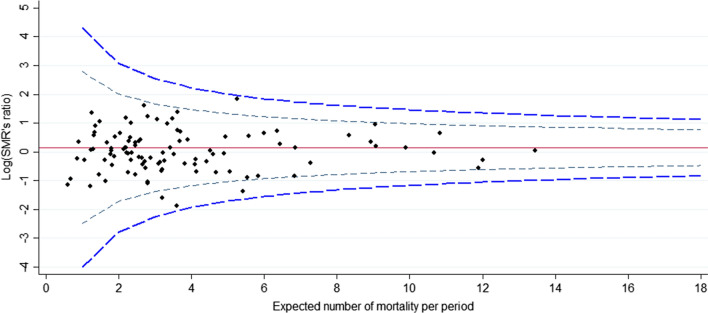
Funnel plot of the change in standardized mortality rates from 2009–2014 to 2015–2018 for 176 hospitals, band limits at 95% (black dash line) and 99.8% (blue dash line)

The funnel plot for the 2009–2014 period is displayed in Fig. 2. In our case, there is no overdispersion because the Winsorized estimator was close to 1. Out of 176 hospitals, 10 were found to lie outside the central 95% region and 7 outside the central 99.8% region (Fig. 2). For the 2015–2018 period, the funnel plot is displayed in Fig. 3. To calculate an overdispersion factor for risk adjusted rates, the Winsorized estimator was 1. Out of 176 hospitals, 6 were found to lie outside the central 95% region and 2 outside the central 99.8% region (Fig. 3). Among the hospitals outliers for the 2015–2018 period, 3 were already outliers for the 2009–2014 period. Figure 4 shows the change in standardized mortality ratios between 2009–2014 and 2015–2018, testing for differences in the overall change (0.024). Out of 176 hospitals, 4 lie outside the central 95% region and 0 lie outside the central 99.8% region (Fig. 4).

## Discussion

While the present study shows that there has been a decrease in mortality following pancreatectomy in recent years, the rate remains higher than what has been reported in the literature. For instance, a meta-analysis conducted in 2015 reported an estimated postoperative mortality rate of 3.1%, with a 95% CI ranging from 2.4 to 3.9% [18].

The graphical analyses using the funnel plot indicate that there has been a slight decrease in outliers over time. However, the SMR ratio shows that only 3 centers improved mortality outcomes and that one center actually worsened the quality of care. The majority of hospitals have remained close to the national benchmark without any real change in the quality of care. It is difficult to find a single explanation for the lack of improvement in the quality of care. However, the number of centers that perform major pancreatic resections is high in France compared to other countries [4, 5], and the number of centers performing pancreatic resection is also high considering the needs of the French population. A study published in 2011 by Finks et al. [4] reported a median annual volume of 16 procedures in the USA, versus only 5–7 for France.

Previous work using the French medico-administrative database has shown that hospitals performing less than 10 pancreatic resections per year have significantly higher postoperative mortality than hospitals that do more than 20 pancreatectomies per year [19]. The authors also found a linear decrease in postoperative mortality depending on volume (9.1%, 8.1% and 5.3% in low, intermediate and high volume centers, respectively) [19]. Other studies have confirmed the influence of activity volume on the quality of care provided by hospitals and surgeons [20, 21].

Some countries have implemented the centralization of complex surgeries in selected hospitals in an attempt to group together the technical skills needed to improve the quality of care [5, 6, 22]. This idea seems attractive, especially if we want to be able to measure hospital performance reliably. Compared with other countries, France offers dispersed care for major interventions such as pancreatic surgery, and most private and public hospitals will perform a pancreatic resection at least once a year. This practice could be one of the reasons that the death rate is higher in France than in other countries [3, 4, 6, 22].

There was no overdispersion of our quality indicator, which can be explained by the quality of the model used [17]. The graphical funnel plot method is a relevant tool for the evaluation of the quality of hospitals. It is rather conservative method for the detection of outliers, with a low probability of wrongly classifying a hospital beyond the limits [16]. Hospitals classified as “worse” have a standardized mortality rate beyond the upper limit of 99.8% of the funnel plot. They have an excess of mortality compared to the national average. On the other hand, establishments classified as “better” have a standardized mortality rate below the limit below 99.8% of the funnel plot. In our work, we are not interested in these establishments. The focus of this study was on teams with excess mortality. This method could easily be used in France for a number of surgical procedures by choosing the most relevant quality indicator. The funnel plots are flexible and minimize the risk of falsely ranking a hospital as an outlier.

The limitations of the study are primarily related to the quality of the data used to develop the risk-adjusted model. The quality of hospital data has improved in France over the last few years, but there is still considerable potential for miscoding. Coding practices vary greatly among institutions, resulting in higher rates of recorded comorbidities in certain hospitals and sub-coding in others. However, the data are representative of French practice. While the results of the present study are based on French data, some of the conclusions may nonetheless be generalizable to other settings.

## Conclusion

Pancreatic surgery is practiced by many centers, and as a result some institutions operate on as little as one patient per year. Over time, the observed improvement in hospital quality was weak. The results of this study suggest that there is a pressing need to reorganize the supply of care for pancreatic surgery in France.
